# Supplementary figures and images for: T6SS-mediated competition by Stenotrophomonas rhizophila shapes seed-borne bacterial communities and seed-to-seedling transmission dynamics
Source: mSystems. 2025 Jul 16;10(8):e00457-25. doi: 10.1128/msystems.00457-25 (PMC12363174; doi:10.1128/msystems.00457-25)

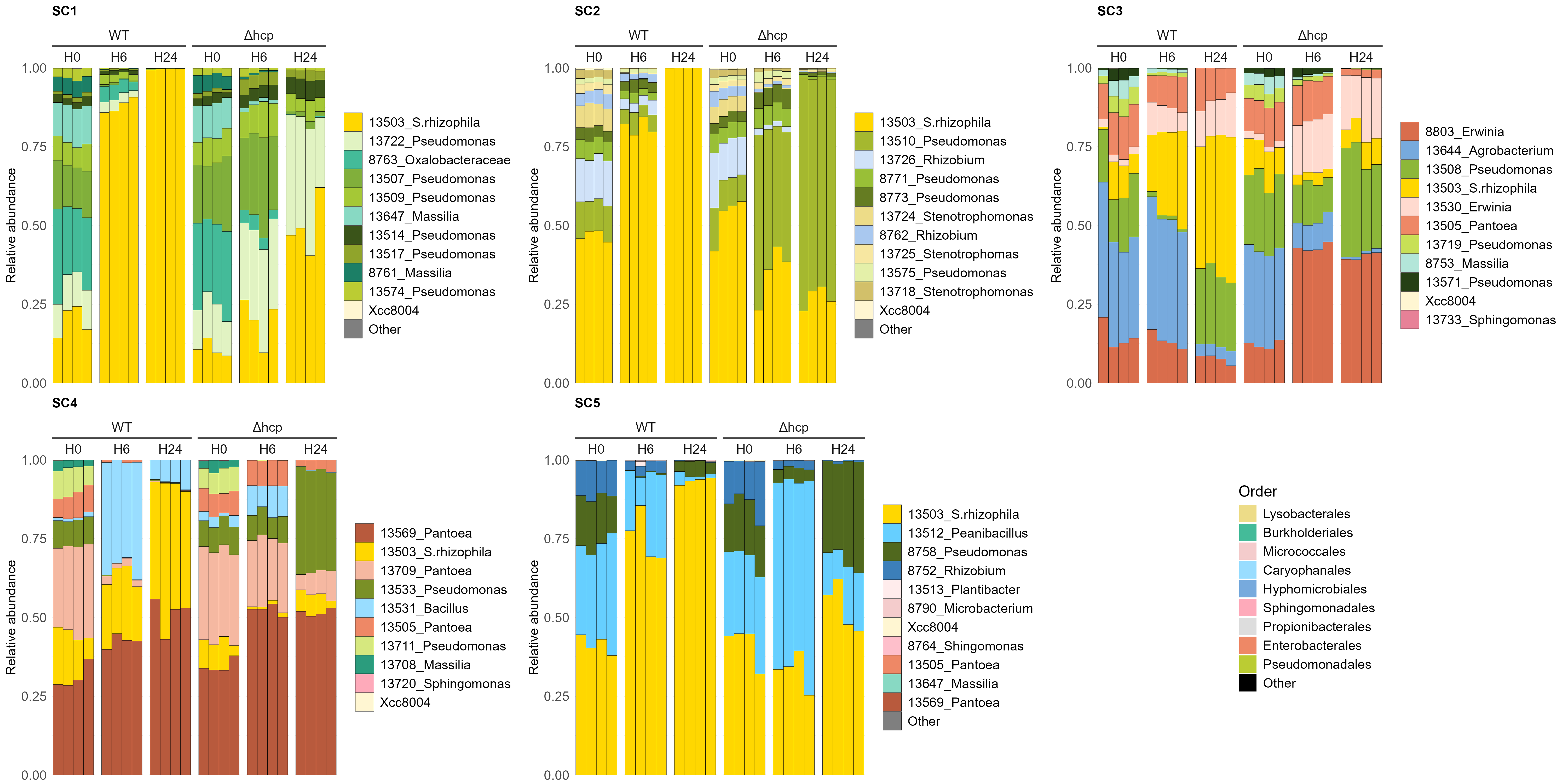

Supplement: Fig. S1 — T6SS impact on the structure of bacterial synthetic communities in vitro. [file msystems.00457-25-s0001.png]

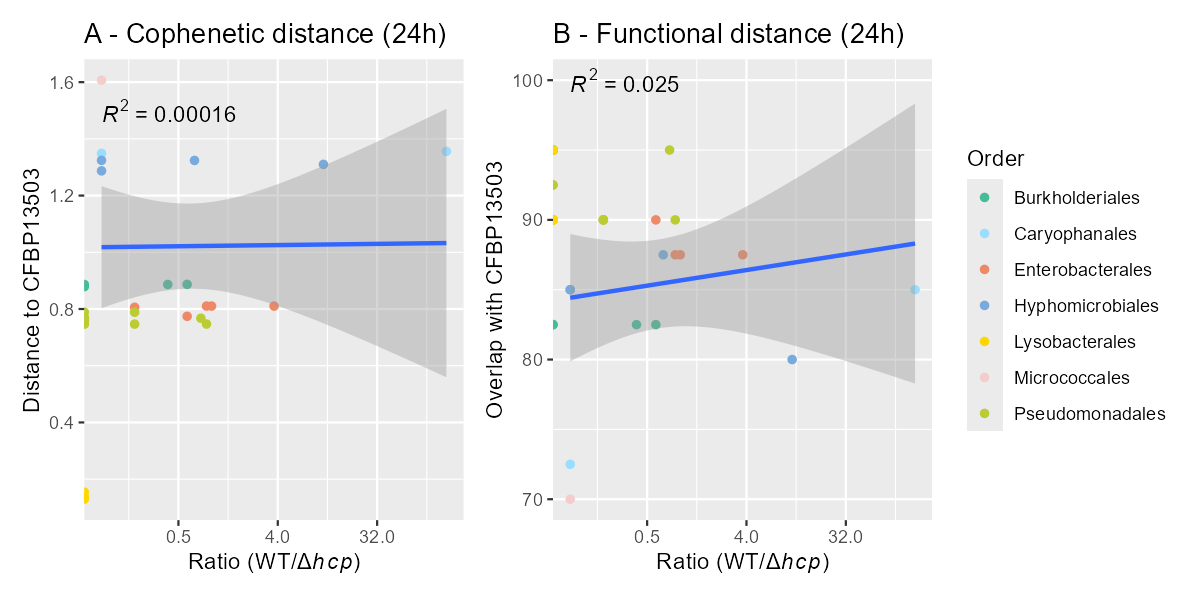

Supplement: Fig. S2 — Relationship between phylogenetic/functional distances and RA at 24 h of confrontation of SynCom members. [file msystems.00457-25-s0002.tiff]

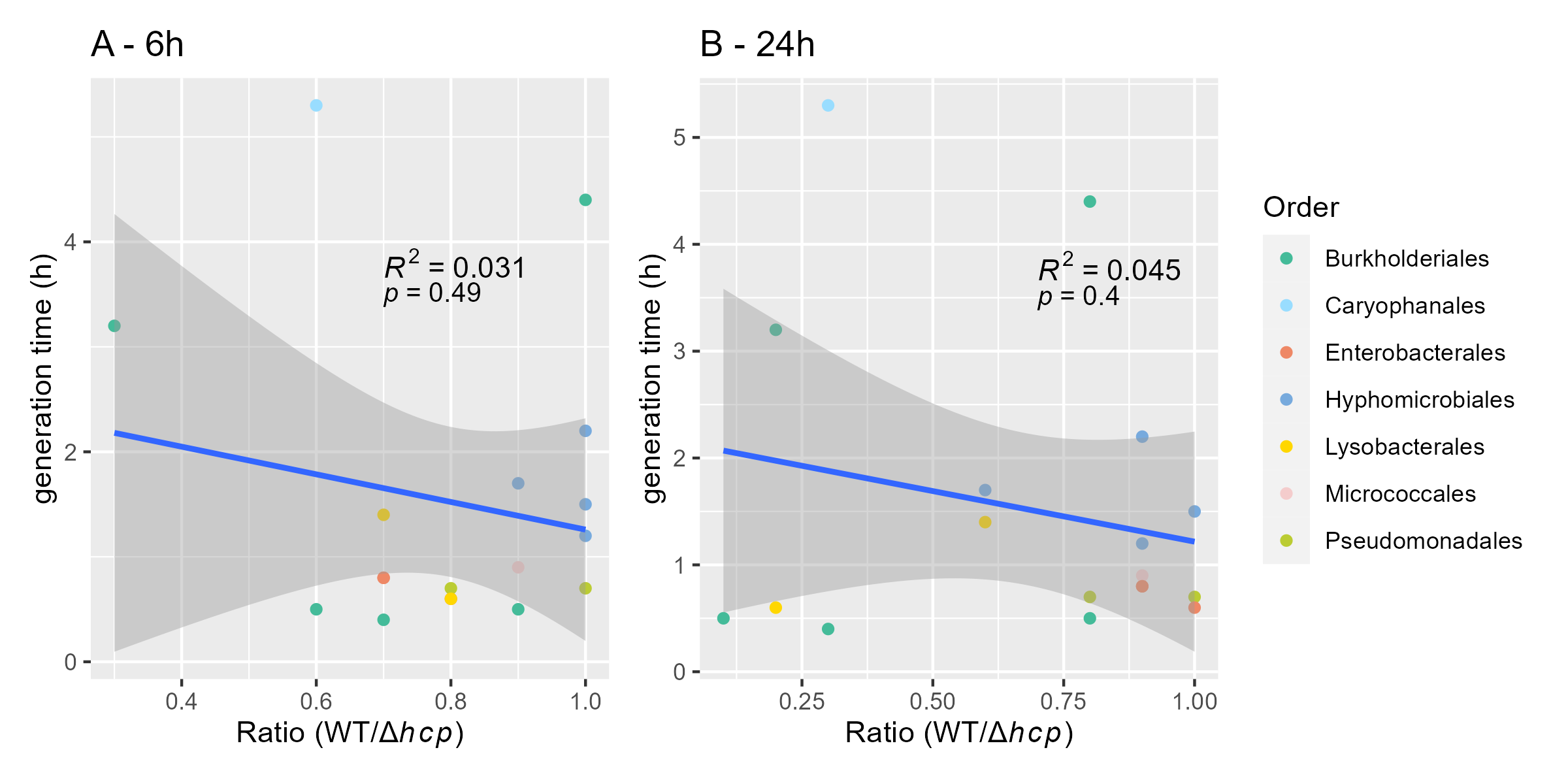

Supplement: Fig. S3 — Relationship between strain sensitivity to T6SS and strain growth rate at 6 and 24 h of confrontation with CFP13503 (WT) and ∆hcp. [file msystems.00457-25-s0003.tiff]

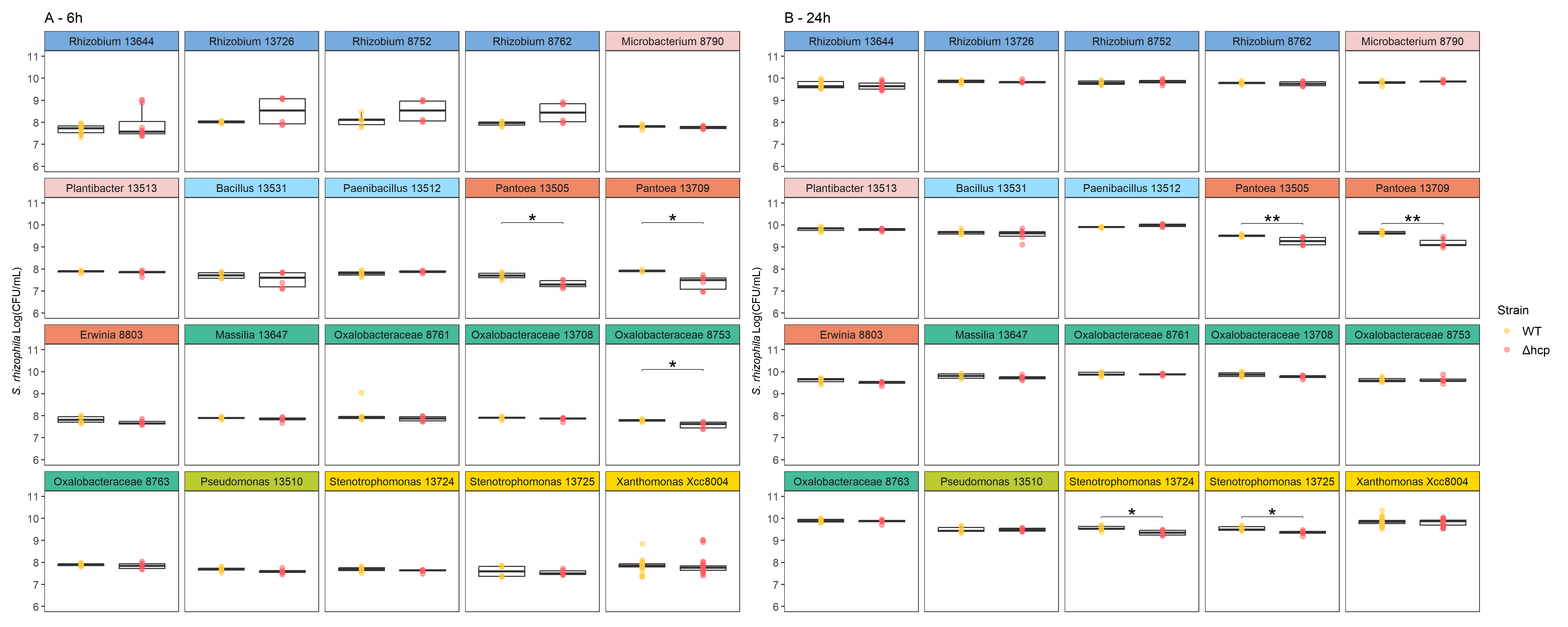

Supplement: Fig. S4 — Population dynamics of S. rhizophila CFBP13503 and T6SS-deficient mutant Δhcp during in vitro confrontation with seed-borne bacterial strains. [file msystems.00457-25-s0004.tiff]

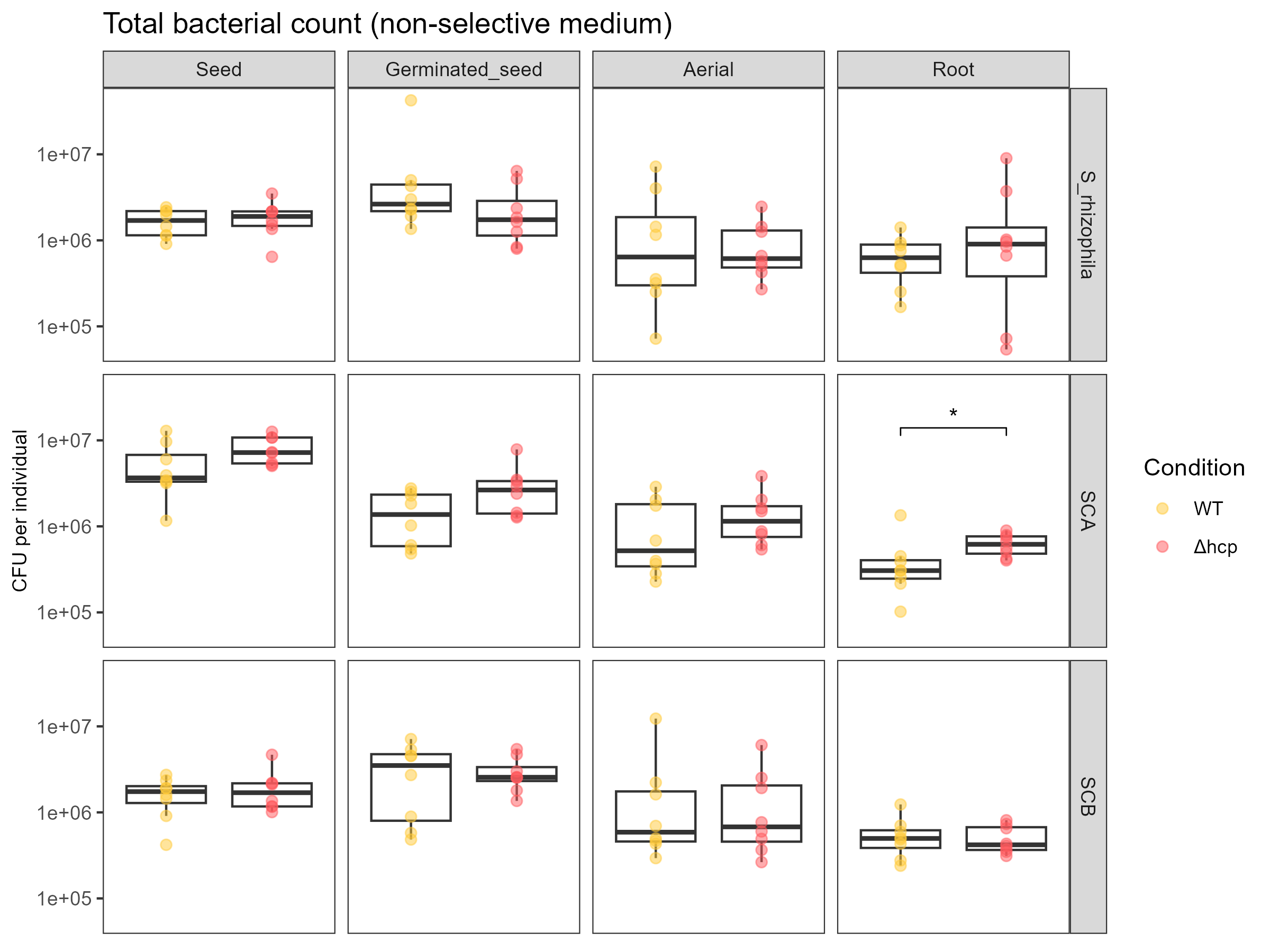

Supplement: Fig. S5 — Abundance of total bacteria during seed-to-seedling transmission. [file msystems.00457-25-s0005.tiff]

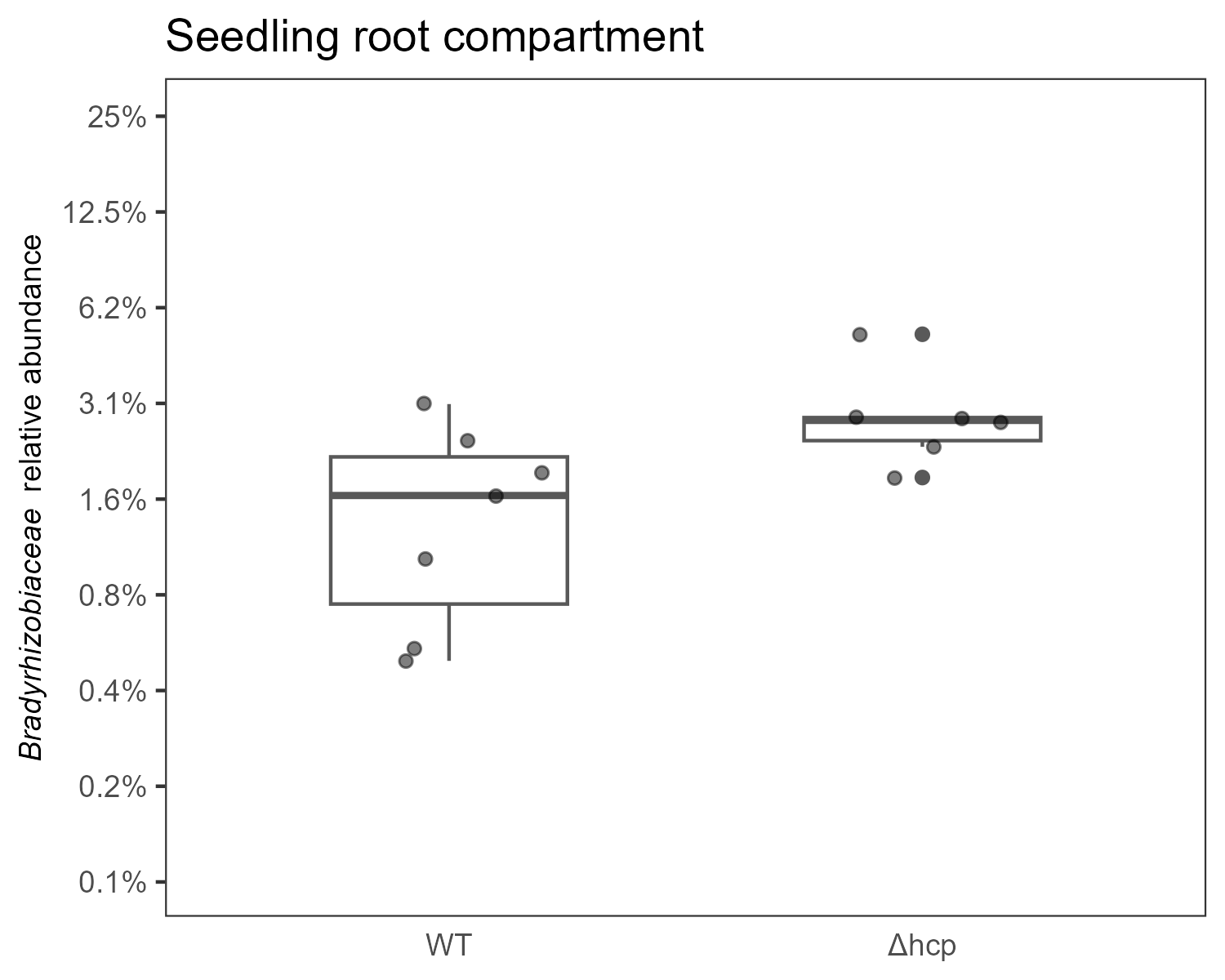

Supplement: Fig. S6 — Relative abundance of ASVs affiliated to Bradyrhizobiaceae in roots following seed inoculations of S. rhizophila CFBP13503 strains. [file msystems.00457-25-s0006.tiff]
