# Supplementary material for: T6SS-mediated competition by Stenotrophomonas rhizophila shapes seed-borne bacterial communities and seed-to-seedling transmission dynamics
Source: mSystems. 2025 Jul 16;10(8):e00457-25. doi: 10.1128/msystems.00457-25 (PMC12363174; doi:10.1128/msystems.00457-25)
Supplement: Supplemental Legends — Legends for Figures S1 to S6 and Tables S1 to S3. [file msystems.00457-25-s0007.docx]

**SUPPLEMENTAL LEGENDS**

**Figure S1.** **T6SS impact on the structure of bacterial synthetic communities *in vitro.***

Relative abundance of bacterial taxa within five different synthetic communities (SynCom) at different confrontation times (0h, 6h, and 24h) while competing with the wild-type strain of *S. rhizophila* CFBP13503 (WT) or the T6SS-deficient mutant Δ*hcp*.

**Figure S2 Relationship between phylogenetic/functional distances and RA at 24h confrontation of SynCom members.** Correlation between changes in relative abundance of SynCom members at 24h and their cophenetic distances to CFBP13503 (**A**). Correlation between changes in relative abundance of SynCom members at 24h and their resource overlap with CFBP13503 (**B**).

**Figure S3. Relationship between strain sensitivity to T6SS and strain growth rate at 6h and 24h of confrontation with CFP13503 (WT) and ∆*hcp*.**

Strain sensitivity is reported as the ratio (LogCFU WT/**∆***hcp*) and growth rate as generation time (h) in TSB1/10.

**Figure S4. Population dynamics of *S. rhizophila* CFBP13503 and T6SS-deficient mutant *Δhcp*** **during *in vitro* confrontation with seed-borne bacterial strains**. *S. rhizophila* populations (CFU.ml^-1^) were monitored after confrontation with rifampicin-resistant strains in TSA10 medium for 6h (A) and 24h (B). Colony-forming units (CFU) were quantified on TSA10 supplemented with spectinomycin and ampicillin. Six replicates are plotted. Statistical analyses were performed using Wilcoxon-Mann-Whitney Test (* p-value < 0.05).

**Figure S5. Abundance of total bacteria during seed to seedling transmission**. Bacterial community size was enumerated on TSA10 medium. The colors represent the initial seed inoculation with *S. rhizophila* CFBP13503 strain WT (yellow) and Δ*hcp* (red). Data are plotted as CFU / individual. Statistical analyses were performed using Wilcoxon-Mann-Whitney test (* p-value < 0.05).

**Figure S6. Relative abundance of ASVs affiliated to *Bradyrhizobiaceae* in roots following seed-inoculations of *S. rhizophila* CFBP13503 strains (WT or T6SS-deficient mutant).** Relative abundance data were log2 transformed for display purposes.

**Table S1. Investigation of T6SS presence within genome sequences of seed and seedling-associated bacterial strains.**

**Table S2. Strains used to build the different SynComs in this study**

**Table S3. Percentage of variance in bacterial phylogenetic composition of SynCom 1 to 5 (SC1-SC5) explained by initial stains (Strain) composition, time of confrontation (6h and 24h, Time) or Strain x Time interaction.**
