# Supplementary material for: T6SS-mediated competition by Stenotrophomonas rhizophila shapes seed-borne bacterial communities and seed-to-seedling transmission dynamics
Source: mSystems. 2025 Jul 16;10(8):e00457-25. doi: 10.1128/msystems.00457-25 (PMC12363174; doi:10.1128/msystems.00457-25)
Supplement: Table S3 — Percentage of variance in the bacterial phylogenetic composition of SC1–SC5 explained by initial strain composition, time of confrontation, or strain × time interaction. [file msystems.00457-25-s0010.docx]

Table S3. **Percentage of variance in bacterial phylogenetic composition of SynCom 1 to 5 (SC1-SC5) explained by initial stains (Strain) composition, time of confrontation (6h and 24h, Time) or Strain x Time interaction.**

| SynCom | Strain | Time | Strain x Time |
| --- | --- | --- | --- |
| SC1 | 36.9 | 30.8 | ns |
| SC2 | 29.2 | 37.4 | 24.8 |
| SC3 | 26.2 | 64.3 | 5.2 |
| SC4 | 38.9 | 35.1 | 18.1 |
| SC5 | 75.1 | 6.2 | 13.5 |
