# Supplementary material for: T6SS-mediated competition by Stenotrophomonas rhizophila shapes seed-borne bacterial communities and seed-to-seedling transmission dynamics
Source: mSystems. 2025 Jul 16;10(8):e00457-25. doi: 10.1128/msystems.00457-25 (PMC12363174; doi:10.1128/msystems.00457-25)
Supplement: Supplementary file 5 [file msystems.00457-25-s0008.docx]

CFBP	Taxonomy	Biosample	Host	Compartmer	Year	Plot	Genome	GC%	CDSs	T6SS	i1	i2	i3	i4a	i4b	i5	iii	Bioproject
7767	Xanthomonas citri pv. fuscans	SAMN06829140	Phaseolus vulgaris	Seed	2009	Cameroun	5297402	65	4516	2		1			1			PRJNA383319
8751	Frigoribacterium	SAMN16237779	Brassica napus Tenor	Seed	2017	IGEPP	3539903	71	3293	0								PRJNA665156
8752	Rhizobium	SAMN16237344	Brassica napus Boston	Seed	2017	IGEPP	4888650	60	4580	0								PRJNA665156
8753	Oxalobacteraceae	SAMN16237354	Brassica napus Boston	Seed	2017	IGEPP	5167451	64	4430	1					1			PRJNA665156
8754	Frigoribacterium	SAMN16237571	Brassica napus Major	Seed	2017	IGEPP	3385781	73	3080	0								PRJNA665156
8755	Oxalobacteraceae	SAMN16237644	Brassica napus Major	Seed	2017	IGEPP	5220625	64	4513	1					1			PRJNA665156
8756	Pantoea agglomerans	SAMN16237653	Brassica napus Major	Seed	2017	IGEPP	5029763	55	4637	2		1	1					PRJNA665156
8757	Aeromicrobium	SAMN16237654	Brassica napus Major	Seed	2017	IGEPP	3757853	71	3670	0								PRJNA665156
8758	Pseudomonas putida group	SAMN16237690	Brassica napus Major	Seed	2017	IGEPP	4663592	62	4189	0								PRJNA665156
8759	Frigoribacterium	SAMN16237713	Brassica napus Mohican	Seed	2017	IGEPP	3401111	71	3141	0								PRJNA665156
8760	Sphingomonas	SAMN16237746	Brassica napus Mohican	Seed	2017	IGEPP	4302246	67	3972	1						1		PRJNA665156
8761	Oxalobacteraceae	SAMN16237762	Brassica napus Tenor	Seed	2017	IGEPP	5211781	64	4452	1					1			PRJNA665156
8762	Rhizobium	SAMN16237769	Brassica napus Tenor	Seed	2017	IGEPP	4436340	57	4279	0								PRJNA665156
8763	Oxalobacteraceae	SAMN16237778	Brassica napus Tenor	Seed	2017	IGEPP	5498755	63	4693	2					2			PRJNA665156
8764	Sphingomonas	SAMN16237800	Brassica napus Tenor	Seed	2017	IGEPP	4345566	66	3880	0								PRJNA665156
8765	Sphingomonas	SAMN16237811	Brassica napus Zorro	Seed	2017	IGEPP	4387101	66	3941	0								PRJNA665156
8766	Frigoribacterium	SAMN16237812	Brassica napus Zorro	Seed	2017	IGEPP	3559033	72	3254	0								PRJNA665156
8767	Erwinia persicina	SAMN16237479	Brassica napus Express	Seed	2017	IGEPP	5182457	55	4829	3		3						PRJNA665156
8768	Pseudomonas fluorescens subgroup	SAMN16237480	Brassica napus Express	Seed	2017	IGEPP	6155614	60	5556	2	1		1					PRJNA665156
8769	Pseudomonas putida group	SAMN16237574	Brassica napus Major	Seed	2017	IGEPP	5709324	60	5245	0								PRJNA665156
8770	Pseudomonas putida group	SAMN16237655	Brassica napus Major	Seed	2017	IGEPP	4654033	62	4125	0								PRJNA665156
8771	Pseudomonas putida group	SAMN16237697	Brassica napus Major	Seed	2017	IGEPP	4765370	62	4232	1	1							PRJNA665156
8772	Pseudomonas	SAMN16237710	Brassica napus Major	Seed	2017	IGEPP	5879355	61	5162	3	1	1			1			PRJNA665156
8773	Pseudomonas putida group	SAMN16237754	Brassica napus Mohican	Seed	2017	IGEPP	4654405	62	4121	0								PRJNA665156
8774	Pantoea agglomerans	SAMN16237802	Brassica napus Zorro	Seed	2017	IGEPP	5464831	55	5207	2		1	1					PRJNA665156
8775	Plantibacter	SAMN16237042	Phaseolus vulgaris Flavert	Flow er	2016	FNAMS – 49	4397458	69	4069	0								PRJNA665156
8776	Pseudomonas fluorescens subgroup	SAMN16237045	Phaseolus vulgaris Flavert	Flow er	2016	FNAMS – 49	6046465	60	5454	3	1	1	1					PRJNA665156
8777	Pseudomonas syringae group	SAMN16237050	Phaseolus vulgaris Flavert	Flow er	2016	FNAMS – 49	6139145	62	5479	2	1		1					PRJNA665156
8778	Pseudomonas fluorescens subgroup	SAMN16237054	Phaseolus vulgaris Flavert	Pods	2016	FNAMS – 49	6013069	60	5290	3	1	1	1					PRJNA665156
8779	Pseudomonas syringae group	SAMN16237056	Phaseolus vulgaris Flavert	Pods	2016	FNAMS – 49	6140205	62	5475	2	1		1					PRJNA665156
8780	Pseudomonas orientalis	SAMN16237064	Phaseolus vulgaris Flavert	Seeds	2016	FNAMS – 49	5804252	60	5109	2	1		1					PRJNA665156
8781	Pseudomonas orientalis	SAMN16237067	Phaseolus vulgaris Flavert	Seeds	2016	FNAMS – 49	5802148	60	5109	2	1		1					PRJNA665156
8782	Xanthomonas campestris pv. campestris	SAMN16237897	Raphanus sativus Flamboyant5	Seed	2014	FNAMS – 49	5076693	65	4257	0								PRJNA665156
8783	Pantoea agglomerans	SAMN16237909	Raphanus sativus Flamboyant5	Flow er	2016	FNAMS – 49	5124033	55	4840	2		1	1					PRJNA665156
8784	Pantoea agglomerans	SAMN16237910	Raphanus sativus Flamboyant5	Flow er	2016	FNAMS – 49	4755237	55	4336	2		1	1					PRJNA665156
8785	Pantoea agglomerans	SAMN16237912	Raphanus sativus Flamboyant5	Flow er	2016	FNAMS – 49	5044125	55	4709	2		1	1					PRJNA665156
8786	Pantoea agglomerans	SAMN16237914	Raphanus sativus Flamboyant5	Seed	2016	FNAMS – 49	4928760	55	4576	1			1					PRJNA665156
8787	Pseudomonas fluorescens subgroup	SAMN16237931	Raphanus sativus Flamboyant5	Seed	2016	FNAMS – 49	6016319	60	5295	3	1	1	1					PRJNA665156
8788	Pseudomonas fluorescens subgroup	SAMN16237973	Raphanus sativus Flamboyant5	Seed	2016	FNAMS – 49	6045815	60	5451	3	1	1	1					PRJNA665156
8789	Pseudomonas fluorescens subgroup	SAMN16238009	Raphanus sativus Flamboyant5	Seed	2016	FNAMS – 49	6046475	60	5453	3	1	1	1					PRJNA665156
8790	Pseudomonas fluorescens subgroup	SAMN16238272	Raphanus sativus Flamboyant5	Seed	2016	FNAMS – 49	3445937	70	3168	0								PRJNA665156
8791	Pantoea agglomerans	SAMN16238273	Raphanus sativus Flamboyant5	Seed	2016	FNAMS – 49	4905192	55	4552	2		1	1					PRJNA665156
8792	Pantoea agglomerans	SAMN16238279	Raphanus sativus Flamboyant5	Seed	2016	FNAMS – 49	5239108	55	4959	2		1	1					PRJNA665156
8793	Pseudomonas orientalis	SAMN16238286	Raphanus sativus Flamboyant5	Seed	2016	FNAMS – 49	5799590	60	5105	2	1		1					PRJNA665156
8794	Microbacterium	SAMN16238307	Raphanus sativus Flamboyant5	Seed	2016	FNAMS – 49	3502222	70	3236	0								PRJNA665156
8795	Erwinia persicina	SAMN16238309	Raphanus sativus Flamboyant5	Seed	2016	FNAMS – 49	5148964	55	4784	1		1						PRJNA665156
8796	Pseudomonas orientalis	SAMN16238321	Raphanus sativus Flamboyant5	Seed	2016	FNAMS – 49	5802756	60	5101	2	1		1					PRJNA665156
8797	Erwinia persicina	SAMN16238323	Raphanus sativus Flamboyant5	Seed	2016	FNAMS – 49	4806053	56	4361	2		2						PRJNA665156
8798	Plantibacter	SAMN16238397	Raphanus sativus Flamboyant5	Seed	2016	FNAMS – 49	4301372	69	3949	0								PRJNA665156
8799	Pseudomonas viridiflava	SAMN16237943	Raphanus sativus Flamboyant5	Seed	2016	FNAMS – 49	6035893	59	5388	2	1		1					PRJNA665156
8800	Pantoea agglomerans	SAMN16238278	Raphanus sativus Flamboyant5	Seed	2016	FNAMS – 49	5117730	55	4806	2	1	1						PRJNA665156
8801	Microbacterium	SAMN16237052	Phaseolus vulgaris Flavert	Flow er	2016	FNAMS – 49	3442739	70	3159	0								PRJNA665156
8802	Pseudomonas viridiflava	SAMN16238274	Raphanus sativus Flamboyant5	Seed	2016	FNAMS – 49	5947296	59	5265	2	1		1					PRJNA665156
8803	Erwinia persicina	SAMN16237911	Raphanus sativus Flamboyant5	Flow er	2016	FNAMS – 49	4897238	56	4491	2		2						PRJNA665156
8804	Plantibacter	SAMN16238287	Raphanus sativus Flamboyant5	Seed	2016	FNAMS – 49	4227001	69	3949	0								PRJNA665156
8977	Pseudomonas colepterorum	SAMN38288732	Phaseolus vulgaris Linex	Seedling	2021	FNAMS – 32	4820021	62	4264	0								PRJNA1041598
8978	Lelliottia	SAMN38288733	Phaseolus vulgaris Vanilla	Seedling	2021	FNAMS – 32	4442944	54	4027	3		2	1					PRJNA1041598
8979	Pseudomonas syringae	SAMN38288734	Phaseolus vulgaris Vanilla	Seedling	2021	FNAMS – 49	6056903	59	5146	1					1			PRJNA1041598
8980	Stenotrophomonas	SAMN38288735	Phaseolus vulgaris Caprice	Seedling	2021	FNAMS – 32	3975215	66	3443	0								PRJNA1041598
8982	Pseudomonas colepterorum	SAMN38288736	Phaseolus vulgaris Caprice	Seed	2021	FNAMS – 32	4754523	62	4164	0								PRJNA1041598
8983	Pseudomonas viridiflava	SAMN38288737	Phaseolus vulgaris Deezer	Seed	2021	FNAMS – 49	5866520	59	5188	2	1		1					PRJNA1041598
8984	Pseudomonas rhizosphaerae	SAMN38288738	Phaseolus vulgaris Deezer	Seedling	2021	FNAMS – 49	4572494	62	4073	0								PRJNA1041598
8985	Pseudomonas viridiflava	SAMN38288739	Phaseolus vulgaris Vanilla	Seed	2021	FNAMS – 49	5852582	59	5155	1					1			PRJNA1041598
8986	Kosakonia	SAMN38288740	Phaseolus vulgaris Linex	Seed	2021	FNAMS – 49	4808856	56	4393	1			1					PRJNA1041598
8987	Leclercia	SAMN38288741	Phaseolus vulgaris Vanilla	Seedling	2021	FNAMS – 32	4836644	56	4496	1		1						PRJNA1041598
8988	Pantoea agglomerans	SAMN38288742	Phaseolus vulgaris Deezer	Seedling	2021	FNAMS – 32	4810088	55	4403	2		1	1					PRJNA1041598
8989	Pantoea agglomerans	SAMN38288743	Phaseolus vulgaris Vanilla	Seedling	2021	FNAMS – 49	4766298	55	4375	2		1	1					PRJNA1041598
8990	Siccibacter turicensis	SAMN38288744	Phaseolus vulgaris Vanilla	Seedling	2021	FNAMS – 32	4131421	58	3760	3	1	1	1					PRJNA1041598
8991	Pseudomonas viridiflava	SAMN38288745	Phaseolus vulgaris Linex	Seedling	2021	FNAMS – 32	5864384	59	5187	2	1		1					PRJNA1041598
8992	Pseudomonas fluorescens subgroup	SAMN38288746	Phaseolus vulgaris Caprice	Seedling	2021	FNAMS – 32	6313504	60	5708	3	1	1	1					PRJNA1041598
8993	Sphingomonas	SAMN38288747	Phaseolus vulgaris Linex	Seedling	2021	FNAMS – 49	3824895	67	3436	0								PRJNA1041598
8994	Stenotrophomonas	SAMN38288748	Phaseolus vulgaris Caprice	Seedling	2021	FNAMS – 32	4250922	67	3720	0								PRJNA1041598
8995	Enterobacter	SAMN38288749	Phaseolus vulgaris Linex	Seedling	2021	FNAMS – 49	5194180	55	4735	1		1						PRJNA1041598
8996	Chryseobacterium	SAMN38288750	Phaseolus vulgaris Vanilla	Seedling	2021	FNAMS – 49	4439189	34	4011	1							1	PRJNA1041598
9000	Pantoea agglomerans	SAMN38288751	Phaseolus vulgaris Flavert	Seedling	2021	FNAMS – 49	4725785	55	4300	2		1	1					PRJNA1041598
9003	Pseudomonas atacamensis	SAMN38288752	Phaseolus vulgaris Contender	Seedling	2021	FNAMS – 32	5859533	60	5218	1		1						PRJNA1041598
9006	Stenotrophomonas rhizophila	SAMN38288753	Phaseolus vulgaris Facila	Seedling	2021	FNAMS – 32	4752770	67	4124	1					1			PRJNA1041598
9009	Bacillus	SAMN38288754	Phaseolus vulgaris Flavert	Seedling	2021	FNAMS – 32	5684090	40	5591	0								PRJNA1041598
9010	Priestia megaterium	SAMN38288755	Phaseolus vulgaris Flavert	Seedling	2021	FNAMS – 32	5857104	37	6029	0								PRJNA1041598
9011	Curtobacterium	SAMN38288756	Phaseolus vulgaris Vezer	Seedling	2021	FNAMS – 49	3616265	71	3424	0								PRJNA1041598
9012	Massilia	SAMN38288757	Phaseolus vulgaris Flavert	Seedling	2021	FNAMS – 49	5989676	65	5153	2			1		1			PRJNA1041598
9014	Bacillus thuringiensis	SAMN38288758	Phaseolus vulgaris Flavert	Seedling	2021	FNAMS – 32	5836202	35	5769	0								PRJNA1041598
9016	Pseudoclavibacter	NA	Phaseolus vulgaris Facila	Seedling	2021	FNAMS – 32	3800658	68	3544	0								#N/A
9017	Sphingomonas	NA	Phaseolus vulgaris Facila	Seedling	2021	FNAMS – 32	4316920	66	3804	0								#N/A
9019	Sphingomonas	SAMN38288759	Phaseolus vulgaris Vezer	Seedling	2021	FNAMS – 32	4245651	66	3796	0								PRJNA1041598
9020	Endobacterium cereale	SAMN38288760	Phaseolus vulgaris Flavert	Seedling	2021	FNAMS – 49	6136518	59	5923	1						1		PRJNA1041598
9021	Sphingomonas	SAMN38288761	Phaseolus vulgaris Vezer	Seedling	2021	FNAMS – 49	4314549	66	3872	0								PRJNA1041598
9023	Microbacterium	SAMN38288762	Phaseolus vulgaris Flavert	Seedling	2021	FNAMS – 49	3657683	68	3530	0								PRJNA1041598
9026	Massilia	SAMN38288763	Phaseolus vulgaris Linex	Seedling	2021	FNAMS – 49	5960194	65	5128	2			1		1			PRJNA1041598
9029	Frigoribacterium	SAMN38288769	Phaseolus vulgaris Flavert	Seedling	2021	FNAMS – 49	3208820	71	2943	0								PRJNA1041598
9030	Frigoribacterium	SAMN38288764	Phaseolus vulgaris Deezer	Seedling	2021	FNAMS – 49	3266158	71	3014	0								PRJNA1041598
9031	Flavobacterium	SAMN38288765	Phaseolus vulgaris Vanilla	Seedling	2021	FNAMS – 49	5602649	34	4794	1							1	PRJNA1041598
9032	Pedobacter	SAMN38288766	Phaseolus vulgaris Vanilla	Seedling	2021	FNAMS – 49	5582692	38	4687	1							1	PRJNA1041598
9033	Rathayibacter festucae	SAMN38288767	Phaseolus vulgaris Linex	Seedling	2021	FNAMS – 32	4341678	72	3923	0								PRJNA1041598
9034	Microbacterium	SAMN38288768	Phaseolus vulgaris Vanilla	Seed	2021	FNAMS – 49	3691531	70	3354	0								PRJNA1041598
13502	Pseudomonas fluorescens subgroup	SAMN09044009	Raphanus sativus Flamboyant5	Seed	2013	FNAMS – 49	6201658	61	5582	1			1					PRJNA454856
13503	Stenotrophomonas rhizophila	SAMN09062466	Raphanus sativus Flamboyant5	Seed	2013	FNAMS – 49	4875264	67	4288	1					1			PRJNA459523
13504	Pseudomonas koreensis subgroup	SAMN09062476	Raphanus sativus Flamboyant5	Seed	2013	FNAMS – 49	6595509	59	5839	3	2			1				PRJNA459522
13505	Pantoea agglomerans	SAMN09062489	Raphanus sativus Flamboyant5	Seed	2014	FNAMS – 49	4932464	55	4512	1			1					PRJNA459521
13506	Pseudomonas fluorescens subgroup	SAMN09062490	Raphanus sativus Flamboyant5	Seed	2014	FNAMS – 49	5914838	61	5354	2	1		1					PRJNA459520
13507	Pseudomonas viridiflava	SAMN09062491	Raphanus sativus Flamboyant5	Seed	2014	FNAMS – 49	5925962	59	5232	2	1		1					PRJNA459519
13508	Pseudomonas koreensis subgroup	SAMN09062492	Raphanus sativus Flamboyant5	Seed	2014	FNAMS – 49	6031678	60	5258	1	1							PRJNA459518
13509	Pseudomonas fluorescens subgroup	SAMN09062824	Raphanus sativus Flamboyant5	Seed	2014	FNAMS – 49	6834697	61	6091	2	1		1					PRJNA459517
13510	Pseudomonas fluorescens subgroup	SAMN09062823	Raphanus sativus Flamboyant5	Seed	2014	FNAMS – 49	6347968	60	5627	2	1		1					PRJNA459516
13511	Erwinia persicina	SAMN09063090	Raphanus sativus Flamboyant5	Seed	2014	FNAMS – 49	5203109	55	4848	2		2						PRJNA459515
13512	Paenibacillus	SAMN09063421	Raphanus sativus Flamboyant5	Seed	2014	FNAMS – 49	5251198	39	4622	0								PRJNA459514
13513	Plantibacter	SAMN09063410	Raphanus sativus Flamboyant5	Seed	2014	FNAMS – 49	4231965	69	3901	0								PRJNA459513
13514	Pseudomonas fluorescens subgroup	SAMN09063676	Raphanus sativus Flamboyant5	Seed	2014	FNAMS – 49	6238853	60	5614	2	1		1					PRJNA459512
13515	Pseudomonas viridiflava	SAMN09064882	Raphanus sativus Flamboyant5	Seed	2014	FNAMS – 49	5869336	59	5208	2	1		1					PRJNA459504
13516	Pantoea agglomerans	SAMN09064883	Raphanus sativus Flamboyant5	Seed	2014	FNAMS – 49	5093661	55	4698	2		1	1					PRJNA459503
13517	Pseudomonas fluorescens subgroup	SAMN09064884	Raphanus sativus Flamboyant5	Seed	2014	FNAMS – 49	5954142	60	5358	2	1		1					PRJNA459502
13528	Pseudomonas fluorescens subgroup	SAMN09064885	Raphanus sativus Flamboyant5	Seed	2014	FNAMS – 49	6043101	60	5311	3	1	1	1					PRJNA459501
13529	Stenotrophomonas rhizophila	SAMN09064919	Raphanus sativus Flamboyant5	Seed	2014	FNAMS – 49	4637306	67	3997	1					1			PRJNA459500
13530	Enterobacter cancerogenus	SAMN09064920	Raphanus sativus Flamboyant5	Seed	2013	FNAMS – 49	4901530	56	4559	3		2	1					PRJNA459499
13531	Bacillus simplex	SAMN16237854	Raphanus sativus Flamboyant5	Seed	2013	FNAMS – 49	5965991	40	5792	0								PRJNA665156
13532	Pantoea agglomerans	SAMN09064921	Raphanus sativus Flamboyant5	Seed	2013	FNAMS – 49	4934344	55	4597	2		1	1					PRJNA459498
13533	Pseudomonas orientalis	SAMN16237857	Raphanus sativus Flamboyant5	Seed	2013	FNAMS – 49	6107913	60	5416	3	1	1	1					PRJNA665156
13566	Pantoea agglomerans	SAMN16237858	Raphanus sativus Flamboyant5	Seed	2014	FNAMS – 49	5123550	55	4807	2		1	1					PRJNA665156
13569	Pantoea agglomerans	SAMN16237903	Raphanus sativus Flamboyant5	Seed	2015	NA	4797171	55	4436	2		1	1					PRJNA665156
13570	Plantibacter	SAMN16237824	Raphanus sativus Flamboyant5	Seed	2013	FNAMS – 49	4149054	69	3870	0								PRJNA665156
13571	Pseudomonas syringae group	SAMN16237876	Raphanus sativus Flamboyant5	Seed	2014	FNAMS – 49	6026070	62	5339	2	1		1					PRJNA665156
13572	Pseudomonas viridiflava	SAMN16237826	Raphanus sativus Flamboyant5	Seed	2013	FNAMS – 49	6020391	59	5349	3	1	1			1			PRJNA665156
13573	Pseudomonas fluorescens subgroup	SAMN16237813	Raphanus sativus Flamboyant5	Seed	2013	FNAMS – 49	6390377	60	5665	3	2		1					PRJNA665156
13574	Pseudomonas syringae group	SAMN16237900	Raphanus sativus Flamboyant5	Seed	2015	NA	6002142	62	5323		1		1					PRJNA665156
13575	Pseudomonas colepterorum	SAMN16237901	Raphanus sativus Flamboyant5	Seed	2015	NA	4805492	62	4267	0								PRJNA665156
13578	Pseudomonas viridiflava	SAMN16237030	Phaseolus vulgaris Crosser (K79184)	Seed	NA	NA	6147017	59	5501	3	2	1						PRJNA665156
13579	Pantoea agglomerans	NA	Phaseolus vulgaris Linex (K79238)	Seed	NA	NA	4832156	55	4462	2		1	1					#N/A
13583	Pantoea agglomerans	SAMN16237032	Phaseolus vulgaris Crosser (K79184)	Seed	NA	NA	4776328	55	4353	2		1	1					PRJNA665156
13593	Pantoea agglomerans	SAMN16237033	Phaseolus vulgaris Flavert (K79240)	Seed	NA	NA	5130088	55	4736	3		1	2					PRJNA665156
13594	Paenibacillus	SAMN16237029	Phaseolus vulgaris Linex (K79238)	Seed	NA	NA	7325832	46	6437	0								PRJNA665156
13595	Bacillus megaterium	SAMN16237039	Phaseolus vulgaris Flavert (K79240)	Seed	NA	NA	5775775	38	5892	0								PRJNA665156
13596	Bacillus megaterium	SAMN16236979	Phaseolus vulgaris Rocdor (K79265)	Seed	NA	NA	6069910	37	6201	0								PRJNA665156
13597	Bacillus	SAMN16237031	Phaseolus vulgaris Linex (K79238)	Seed	NA	NA	5441368	41	5220	0								PRJNA665156
13599	Pseudomonas colepterorum	SAMN16237062	Phaseolus vulgaris Flavert	Seed	2016	FNAMS – 49	4843938	62	4277	0								PRJNA665156
13600	Pantoea agglomerans	SAMN16236980	Phaseolus vulgaris Rocdor (K79265)	Seed	NA	NA	4885829	55	4470	2		1	1					PRJNA665156
13601	Pseudomonas syringae group	SAMN16237043	Phaseolus vulgaris Flavert	Flow er	2016	FNAMS – 49	6149110	62	5491	2	1		1					PRJNA665156
13602	Pseudomonas putida group	SAMN16236977	Phaseolus vulgaris Rocdor (K79265)	Seed	NA	NA	4779684	62	4251	0								PRJNA665156
13603	Sphingomonas	SAMN16237060	Phaseolus vulgaris Flavert	Seed	2016	FNAMS – 49	4395607	65	3939	0								PRJNA665156
13604	Pseudomonas fluorescens subgroup	SAMN16237065	Phaseolus vulgaris Flavert	Seed	2016	FNAMS – 49	6242560	60	5614	2	1		1					PRJNA665156
13605	Frigoribacterium	SAMN16237059	Phaseolus vulgaris Flavert	Seed	2016	FNAMS – 49	3326223	71	3094	0								PRJNA665156
13611	Pseudomonas fluorescens subgroup	SAMN16237055	Phaseolus vulgaris Flavert	Pods	2016	FNAMS – 49	6046369	60	5448	3	1	1	1					PRJNA665156
13615	Pseudomonas colepterorum	SAMN16237070	Phaseolus vulgaris Flavert	Seeds	2016	FNAMS – 49	4842123	62	4282	0								PRJNA665156
13616	Pantoea agglomerans	SAMN16237057	Phaseolus vulgaris Flavert	Seeds	2016	FNAMS – 49	4780294	55	4376	2		1	1					PRJNA665156
13617	Microbacterium	SAMN16237041	Phaseolus vulgaris Flavert	Flow er	2016	FNAMS – 49	3559858	71	3281	0								PRJNA665156
13644	Rhizobium	SAMN16237137	Brassica napus Aviso	Seed	2017	IGEPP	5086082	57	4731	1						1		PRJNA665156
13647	Massilia	SAMN16237783	Brassica napus Tenor	Seed	2017	IGEPP	6073959	65	5126	2			1		1			PRJNA665156
13649	Pseudomonas fluorescens subgroup	SAMN16237913	Raphanus sativus Flamboyant5	Flow er	2016	FNAMS – 49	6315997	60	5711	3	1	1	1					PRJNA665156
13700	Pseudomonas fluorescens subgroup	SAMN16237051	Phaseolus vulgaris Flavert	Flow er	2016	FNAMS – 49	6020185	60	5337	3	1	1	1					PRJNA665156
13701	Pseudomonas orientalis	SAMN16237071	Phaseolus vulgaris Flavert	Seeds	2016	FNAMS – 49	5766540	60	5067	2	1		1					PRJNA665156
13702	Pseudomonas fluorescens subgroup	SAMN16237046	Phaseolus vulgaris Flavert	Flow er	2016	FNAMS – 49	6019882	60	5300	3	1	1	1					PRJNA665156
13703	Pseudomonas orientalis	SAMN16237069	Phaseolus vulgaris Flavert	Seed	2016	FNAMS – 49	5804470	60	5113	2	1		1					PRJNA665156
13706	Sphingomonas	SAMN16237122	Brassica napus Astrid	Seed	2017	IGEPP	4753019	66	4265	0								PRJNA665156
13707	Frigoribacterium	SAMN16237123	Brassica napus Astrid	Seed	2017	IGEPP	3554678	73	3269	0								PRJNA665156
13708	Oxalobacteraceae	SAMN16237124	Brassica napus Astrid	Seed	2017	IGEPP	5310716	64	4577	1					1			PRJNA665156
13709	Pantoea agglomerans	SAMN16237131	Brassica napus Astrid	Seed	2017	IGEPP	5248093	55	4996	2		1	1					PRJNA665156
13710	Pseudomonas putida group	SAMN16237132	Brassica napus Astrid	Seed	2017	IGEPP	4671763	62	4146	0								PRJNA665156
13711	Pseudomonas	SAMN16237133	Brassica napus Aviso	Seed	2017	IGEPP	6192813	60	5442	3	1	1			1			PRJNA665156
13712	Frigoribacterium	SAMN16237134	Brassica napus Aviso	Seed	2017	IGEPP	3155852	71	2911	0								PRJNA665156
13713	Pseudomonas fluorescens subgroup	SAMN16237135	Brassica napus Aviso	Seed	2017	IGEPP	6833365	60	6129	5	2		3					PRJNA665156
13714	Sphingomonas	SAMN16237136	Brassica napus Aviso	Seed	2017	IGEPP	4419935	66	3950	0								PRJNA665156
13715	Pseudomonas	SAMN16237138	Brassica napus Aviso	Seed	2017	IGEPP	6196569	60	5438	3	1	1			1			PRJNA665156
13716	Pseudomonas fluorescens subgroup	SAMN16237140	Brassica napus Aviso	Seed	2017	IGEPP	7815059	59	7179	5	2		3					PRJNA665156
13717	Rhizobium	SAMN16237141	Brassica napus Aviso	Seed	2017	IGEPP	5158824	57	4777	1						1		PRJNA665156
13718	Stenotrophomonas	SAMN16237142	Brassica napus Aviso	Seed	2017	IGEPP	4202066	66	3684	0								PRJNA665156
13719	Pseudomonas putida group	SAMN16237175	Brassica napus Boston	Seed	2017	IGEPP	5697150	61	5297	1	1							PRJNA665156
13720	Sphingomonas	SAMN16237345	Brassica napus Boston	Seed	2017	IGEPP	3692396	66	3327	0								PRJNA665156
13721	Massilia	SAMN16237342	Brassica napus Boston	Seed	2017	IGEPP	6071750	65	5129	2			1		1			PRJNA665156
13722	Pseudomonas fluorescens subgroup	SAMN16237190	Brassica napus Boston	Seed	2017	IGEPP	6208084	61	5653	2	1		1					PRJNA665156
13723	Pseudomonas lutea group	SAMN16237352	Brassica napus Boston	Seed	2017	IGEPP	5760407	60	5089	3	1	1	1					PRJNA665156
13724	Stenotrophomonas	SAMN16237355	Brassica napus Boston	Seed	2017	IGEPP	3959299	67	3423	0								PRJNA665156
13725	Stenotrophomonas	SAMN16237356	Brassica napus Colvert	Seed	2017	IGEPP	4180738	66	3656	0								PRJNA665156
13726	Rhizobium	SAMN16237357	Brassica napus Colvert	Seed	2017	IGEPP	4933550	60	4657	0								PRJNA665156
13727	Pseudomonas putida group	SAMN16237372	Brassica napus Colvert	Seed	2017	IGEPP	4608063	62	4090	0								PRJNA665156
13728	Sphingomonas	SAMN16237374	Brassica napus Colvert	Seed	2017	IGEPP	4411320	65	4033	0								PRJNA665156
13729	Frigoribacterium	SAMN16237389	Brassica napus Colvert	Seed	2017	IGEPP	3556539	72	3236	0								PRJNA665156
